# Characterizing myocardial edema and fibrosis in hypertensive crisis with cardiovascular magnetic resonance imaging

**DOI:** 10.1038/s41598-024-74099-9

**Published:** 2024-10-09

**Authors:** Mohammed A. Talle, Pieter-Paul S. Robbertse, Anton F. Doubell, Sa’ad Lahri, Philip G. Herbst

**Affiliations:** 1 Division of Cardiology, Department of Medicine, Faculty of Medicine, and Health Sciences, Stellenbosch University and Tygerberg Hospital, 1 Francie van Zijl Ave, Bellville, Cape Town, 7505 South Africa; 2 Department of Medicine, Faculty of Clinical Sciences, College of Medical Sciences, University of Maiduguri and University of Maiduguri Teaching Hospital, Maiduguri, 600004 Nigeria; 3 Division of Emergency Medicine, Department of Medicine, Faculty of Medicine and Health Sciences, Stellenbosch University and Tygerberg Hospital, Cape Town, 7505 South Africa

**Keywords:** Hypertensive crisis, Hypertensive emergency, Hypertensive urgency, Left ventricular hypertrophy, Myocardial, Tissue characteristics, Cardiovascular magnetic resonance, Cardiology, Hypertension

## Abstract

A hypertensive crisis is associated with an increased risk of cardiovascular events. Although altered cardiac structure, function, and myocardial architecture on cardiovascular magnetic resonance (CMR) have been associated with increased adverse events in hypertensive patients, the studies did not include patients with hypertensive crisis. Our study aimed to determine myocardial tissue characteristics in patients with hypertensive crisis using CMR imaging. Participants underwent comprehensive CMR imaging at 1.5T. The imaging protocol included cine-, T2-weighted-, contrasted- and multi-parametric mapping images. Blood and imaging biomarkers were compared in hypertensive emergency and hypertensive urgency. Predictors of myocardial edema was assessed using linear regression. The predictive value of T1- and T2 mapping for identifying hypertensive emergency (from urgency) was assessed with receiver operator characteristics curves. Eighty-two patients (48.5 ± 13.4 years, 57% men) were included. Hypertensive emergency constituted 78%. Native T1 was higher in patients with LVH compared to those without (1056 ± 33 vs. 1013 ± 40, *P* < 0.001), and tended to be higher in hypertensive emergency than urgency (1051 ± 37 vs. 1033 ± 40, *P* = 0.077). T2-w signal intensity (SI) ratio and T2 mapping values were higher in hypertensive emergency (1.5 ± 0.2 vs. 1.4 ± 0.1, *P* = 0.044 and 48 ± 2 vs. 47 ± 2, *P* = 0.004), and in patients with than without LVH (1.5 ± 0.2 vs. 1.4 ± 0.1, *P* = 0.045 and *P* = 0.030). A trend for higher extracellular volume was noted in hypertensive emergency compared to urgency (25 ± 4 vs. 22 ± 3, *P* = 0.050). Native T1 correlated with T2 mapping (rs = 0.429, *P* < 0.001), indexed LV mass (rs = 0.493, *P* < 0.001), cardiac troponin (rs = 0.316, *P* < 0.001) and NT-proBNP (rs = 0.537, *P* < 0.001), while T2 correlated with cardiac troponin (rs = 0.390, *P* < 0.001), and NT-proBNP (rs = 0.348, *P* < 0.001). Non-ischemic LGE pattern occurred in 59% and was 21% more prevalent in the hypertensive emergency group (*P* = 0.005). Our findings demonstrate that hypertensive crisis is associated with distinct myocardial tissue alterations, including increased myocardial edema and fibrosis, as detected on CMR. Patients with hypertensive emergency had a higher degree of myocardial oedema than hypertensive urgency. Further research is necessary to explore the prognostic value of these findings.

**Supplementary Information:**

The online version contains supplementary material available at 10.1038/s41598-024-74099-9.

## Introduction

Hypertensive crisis is a common indication for emergency room visit, affecting 1–2% of hypertensive patients in a lifetime^1^. Despite advances in the management of hypertension and related complications, the incidence of hypertensive crises remains unchanged over the decades, especially in low-and middle income countries^2,3^. Hypertensive crises encompass two distinct clinical conditions: hypertensive urgency and hypertensive emergency. Hypertensive urgency involves severely elevated blood pressure without acute target organ damage, whereas hypertensive emergency is characterized by severe hypertension with evidence of acute organ damage, such as to the heart, brain, or kidneys^4^. Differentiating between these conditions is critical as it influences the urgency and type of medical intervention required.

Presentation with a hypertensive crises have been associated with major adverse cardiovascular events resulting in increased morbidity and mortality^5,6^. Factors associated with poor prognosis include increased levels of cardiac biomarkers, as well as the remodelling associated with long standing hypertension and hypertensive heart disease^7–9^. Prior studies have shown that cardiac biomarkers like cardiac troponin and brain natriuretic peptide are elevated in acute cardiovascular conditions reflecting myocardial injury and increased cardiac filling pressures respectively^8,10^. Perivascular fibrosis and microvascular dysfunction associated with hypertensive cardiac remodelling predispose to myocardial ischemia, especially subendocardial ischemia^11,12^. This can be exacerbated by an acute hypertensive crisis to result in both cytogenic (intracellular) edema, and vasogenic (extracellular interstitial) edema when prolonged^11^. A link has been established between acute arterial hypertension and myocardial edema in experimental models^13^. Both myocardial edema and fibrosis has also been demonstrated in aortic stenosis, a prototype cardiac pressure-loading condition^14,15^. However, previous studies on myocardial tissue characterization in hypertension did not include patients with hypertensive crisis.

Cardiovascular magnetic resonance (CMR) imaging is an advanced modality capable of non-invasively assessing myocardial tissue characteristics, including fibrosis and edema, thus providing detailed insights into myocardial health that other imaging techniques may not offer. These features are essential for understanding the extent of myocardial damage, which may differ between hypertensive urgency and emergency due to varying levels of cardiac stress and damage. Similar to the elevation of cardiac troponin observed in acute stroke patients, which indicates myocardial injury^10^, patients with hypertensive emergency may exhibit distinct myocardial changes detectable by CMR. This underscores the potential for CMR to differentiate between the cardiac impact of hypertensive urgency and hypertensive emergency. This study aims to utilize CMR to explore these differences, thereby improving our understanding of the pathophysiology and clinical management of hypertensive urgency and emergency.

## Methods

### Study population

Patients with hypertensive crisis were recruited prospectively from the medical emergency department of Tygerberg hospital in the Western Cape Province of South Africa based on initial clinical assessments. Eligibility was determined according to established criteria for hypertensive urgency and emergency^4^. Inclusion criteria included adults aged ≥ 18 years with systolic blood pressure ≥ 180 mmHg and/or diastolic blood pressure ≥ 110 mmHg. Exclusion criteria included hypertensive disorders of pregnancy, altered level of consciousness, refusal of consent, age under 18 years, and contraindications to CMR.

The patients were categorized as having a hypertensive emergency where there was evidence of acute hypertension-mediated organ damage (HMOD) or hypertensive urgency where the presentation was not associated with acute HMOD^4^. The group with a hypertensive emergency was subclassified into acute pulmonary edema, myocardial infarction, and neurological emergencies based on the type of acute HMOD^4^. All participants had guideline-directed baseline investigations, including high-sensitivity cardiac troponin T (hs cTnT) and NT-proBNP. The study was approved by the Health and Research Ethics Committee of Stellenbosch University (Approval No. S19/07/117 (PhD)). Written informed consent was obtained from all participants after a comprehensive explanation of the study objectives, procedures, and potential risks. Patient confidentiality was protected by de-identifying all data and securely storing records according to institutional guidelines. The Declaration of Helsinki was adhered to.

### Cardiac magnetic resonance imaging

CMR imaging was performed using a 1.5T scanner (MAGNETOM Avanto, Siemens Healthcare, Erlangen, Germany) within 48 h of presentation to Tygerberg Hospital. The scanning protocol included balanced steady-state free precession (bSSFP) cine (TR/TE: 47.55/1.31ms, matrix size:205 × 256); T2-weighted short-TI inversion recovery (T2-w STIR, TR/TE: 2423/51ms, matrix: 277 × 370, voxel size: 1.5 × 1.5 × 8mm^3^); T1 mapping (native and post-contrast) using Modified Look-Locker Inversion recovery (MOLLI) sequences^16^ (native T1 mapping-TR/TE:289.7/1.33ms, voxel size: 1.5 × 1.5 × 8.0mm^3^; post-contrast T1 mapping- TR/TE:343.16/1.18ms, voxel size: 1.5 × 1.5 × 8.0mm^3^); T2 mapping using bSSFP readout (TR/TE:241.1/1.23ms, voxel size: 2.0 × 2.0 × 8.0mm^3^); and late gadolinium enhancement (LGE) imaging using phase-sensitive inversion-recovery (PSIR) sequences, 10–12 min following intravenous administration of 0.2 ml/kg of gadolinium-based contrast (Gadovist, Bayer Pharma AG, Germany)^17^. The inversion time for nulling the myocardium was individualized using the TI-scout sequence and optimized based on subsequent images obtained. The images were acquired in various orientations, including two-, four- and three-chamber long axis views (for cine and LGE); and a short axis stack covering the entire left ventricle from base to apex (for cine, T2-w STIR, mapping, and LGE). In our unit, we routinely obtain the short-axis stack for LGE in two encoding directions by phase swapping, and additional orthogonal views of LGE images were obtained where necessary.

### Image analysis

MAT performed post-processing of images unblinded using commercially available software (Circle Cardiovascular Imaging, version 5.13.10 [2678] Calgary, Canada). Epicardial and endocardial contours were drawn in the end-diastolic and end-systolic phases of the ventricles to determine cardiac volumes, mass, and LV ejection fraction^18^. Papillary muscles were excluded from the LV cavity and included in LV mass. The biplane area-length method was used to determine the left atrial volume (LAV) and the LAV index derived by dividing LAV by body surface area (BSA). Left ventricular hypertrophy was defined as elevated LV mass indexed to BSA for sex, using published reference values^18^. Similarly, the published CMR reference values for age and sex were used in defining impaired systolic function and abnormal cardiac volumes. The reproducibility of morphological and functional CMR parameters in our unit has been published previously^19^.

For T2-w signal intensity (SI) and parametric mappings, endocardial and epicardial contours were drawn in the basal-, mid-, and apical short-axis slices of the relevant sequence from which the segmental and global T2-w SI, native T1 time and T2 time were determined^17^. Epicardial and endocardial contours were carefully drawn to exclude the blood pool and epicardial fat, applying an offset of 20% and 10% from the epicardial and endocardial borders, respectively. To determine the T2-w SI ratio (myocardial: skeletal muscle), a region of interest was drawn in the serratus anterior muscle of the same slice containing endocardial and epicardial contouring for myocardial T2-w SI, and the ratio calculated as T2-w SI = SI_myocardium_/SI_skeletal muscle_, as previously described^20^. Extracellular volume fraction was determined using native and postcontrast myocardial and blood pool T1 time and haematocrit measured within 24 h of undergoing CMR imaging^21,22^. We have previously published the inter-reader reproducibility of T2-w SI ratio and multi-parametric mapping, demonstrating the interclass correlation ranging from good to excellent^23^.

Late gadolinium enhancement was assessed qualitatively. The LGE images were interpreted in conjunction with other sequences analyzing the same cardiac segment/region, and signals were excluded if considered to be blood pool or epicardial fat. Late gadolinium enhancement was deemed present when visualized in 2 orthogonal planes or 2 adjacent slices, and was classified into ischemic LGE pattern (when subendocardial or transmural consistent with coronary distribution) or non-ischemic LGE pattern (mid myocardial, focal, patchy, right ventricular [RV] insertion point)^24,25^. Myocarditis was diagnosed using the modified Lake-Louis criteria^26^. Areas of wall motion abnormality with subendocardial or transmural LGE (with or without microvascular obstruction), and increased T2 time on mapping (and/or increased STIR signal) were considered acute myocardial infarction^27^.

### Statistical analysis

Statistical analysis was performed using SPSS version 28.0 (IBM, Armonk, NY). Continuous variables were expressed as mean ± standard deviation or median (interquartile range) and compared using Student’s t-test, Mann-Whitney U test, and One-way ANOVA as appropriate. Categorical variables were compared using Chi-square or Fisher’s exact test. Adjustments for multiple comparisons were made using the Bonferroni correction. The correlation of continuous variables was determined using Pearson’s or Spearman’s correlation (two-tailed P-value) as appropriate, while simple linear regression analysis was used to determine categorical variables associated with T2 relaxation time. GraphPad Prism version 6.04 for MacOS (GraphPad Software, www.graphpad.com) was used in making scatter plots. A two-tailed P value of < 0.05 was considered significant for all analyses.

## Results

### Clinical and demographic characteristics

**Fig. 1:**
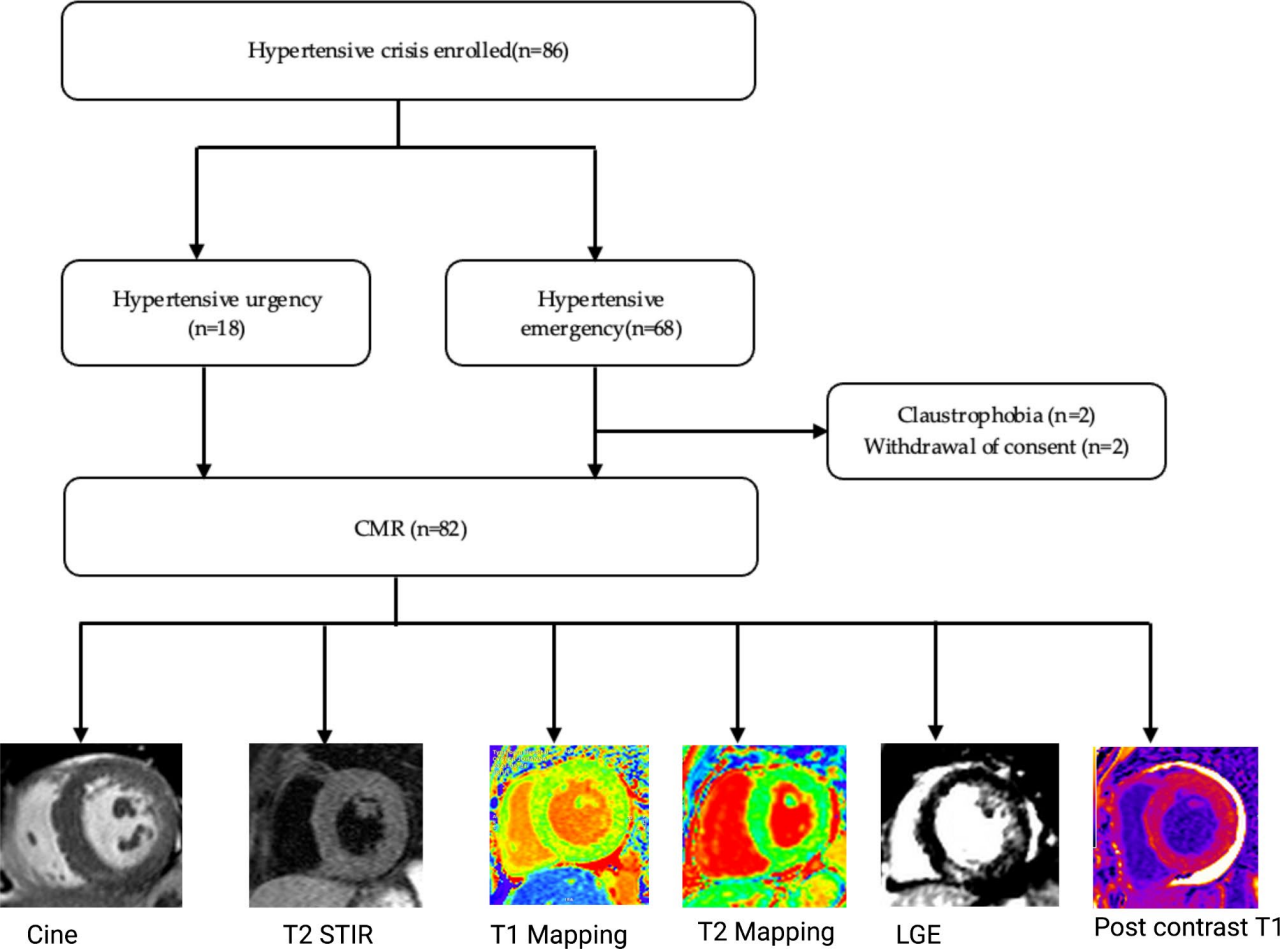
Study flow chart. *CMR* cardiovascular magnetic resonance imaging, *LGE* late gadolinium enhancement, *RV* right ventricular, *STIR* short tau inversion recovery.

Eighty-two patients with hypertensive crisis (48.5 ± 13.4 years, 57% males) underwent CMR (Fig. 1). Table 1 shows the baseline clinical characteristics and CMR imaging profile of the participants. Majority of the patients had hypertensive emergency (78%). The mean age of the total cohort was 48.5 ± 13.4 years, and age did not differ significantly between the hypertensive emergency and hypertensive urgency (*P* = 0.972). Systolic blood pressure is comparable between the hypertensive emergency and hypertensive urgency (*P* = 0.827). However, diastolic blood pressure was significantly higher hypertensive emergency than hypertensive urgency (*P* = 0.032).

Cardiac complications comprising acute pulmonary edema and acute myocardial infarction constituted the majority of acute HMOD (65.7%), and underscores the relevance of cardiac imaging including CMR in the evaluation of patients with hypertensive emergency. Neurological complications (composite of intracranial hemorrhage, ischemic stroke, transient ischemic attack, and hypertensive encephalopathy) occurred in 34.4%. We had previously published the clinical profile including comorbidities of the study participants^28^.

### CMR characteristics

#### Morphology and function

Table 1 illustrates the comparison of CMR findings between hypertensive emergency and hypertensive urgency. The indexed LV mass was 35 g/m^2^ higher in the hypertensive emergency group compared to the hypertensive urgency (*P* < 0.001). Similarly, LVH was 45% more prevalent in hypertensive emergency compared to hypertensive urgency (*P* < 0.001). Increased LV mass and prevalence of LVH in hypertensive emergency reflects the significantly increased DBP than in hypertensive urgency. Systolic function measured using LV ejection fraction was decreased in the hypertensive emergency group, suggesting more advanced cardiac remodelling than hypertensive urgency (50% vs. 68%, *P* < 0.001).

**Table 1:** Clinical, laboratory and cardiac magnetic resonance imaging profile.

Variable	All cases (*n* = 82)	Hypertensive urgency (*n* = 18)	Hypertensive emergency (*n* = 64)	P*
Clinical and laboratory				
Age	48.5 ± 13.4	48.4 ± 15	48.6 ± 13	0.958
Females	35 (43)	13 (72)	22 (34)	0.006
Systolic blood pressure	217 ± 28	216 ± 26	218 ± 29	0.827
Diastolic blood pressure	128 ± 20	119 ± 16	130 ± 20	0.021
Creatinine (µmol/L)	104 (84 to 130)	83 (68 to 97)	113 (93 to 181)	< 0.001
Hemoglobin (g/L)	13.8 ± 2.1	13.6 ± 1.8	13.7 ± 2.2	0.827
hs cTnT (ng/L)	25 (12 to 127)	11 (7 to 13)	41 (17 to 162)	< 0.001
NT-proBNP (ng/L)	377 (64 to 1566)	90 (35 to 302)	528 (113 to 1939)	0.008
Cardiovascular magnetic resonance imaging				
Indexed LV EDV (ml/m^2^)	74 (63 to 101)	63 (59 to 71)	86 (67 to 110)	< 0.001
Indexed LV ESV (ml/m^2^)	32 (22 to 58)	20 (15 to 28)	39 (25 to 69)	< 0.001
LV ejection fraction (%)	54 ± 16	68 ± 8	50 ± 15	< 0.001
Indexed LV mass (g/m^2^)	107 (83 to 140)	80 (66 to 95)	115 (98 to 153)	< 0.001
LVH, n(%)	65 (79)	8 (44)	57 (89)	< 0.001
Global T2-w SI ratio	1.4 ± 0.2	1.4 ± 0.1	1.5 ± 0.2	0.044
Basal T2-w SI ratio	1.5 ± 0.3	1.3 ± 0.2	1.5 ± 0.3	0.022
Midventricular T2-w SI ratio	1.4 ± 0.2	1.4 ± 0.2	1.5 ± 0.2	0.435
Apical T2-w SI ratio	1.4 ± 0.2	1.4 ± 0.2	1.4 ± 0.2	0.821
Global native T1 (ms)	1047 ± 38	1033 ± 40	1051 ± 37	0.077
Basal native T1 (ms)	1053 ± 36	1039 ± 39	1057 ± 34	0.063
Midventricular native T1 (ms)	1047 ± 44	1030 ± 44	1050 ± 44	0.082
Apical native T1 (ms)	1041 ± 44	1025 ± 46	1045 ± 43	0.096
Global T2 (ms)	49 ± 2	48 ± 2	49 ± 2	0.069
Basal T2 (ms)	48 ± 2	47 ± 2	48 ± 2	0.004
Midventricular T2 (ms)	49 ± 3	48 ± 2	49 ± 3	0.060
Apical T2 (ms)	48 ± 5	48 ± 3	49 ± 3	0.157
Global ECV (%)	24 ± 4	23 ± 4	25 ± 4	0.202
Basal ECV (%)	24 ± 4	23 ± 4	25 ± 4	0.152
Midventricular ECV (%)	24 ± 4	22 ± 3	25 ± 4	0.051
Apical ECV (%)	27 ± 5	24 ± 7	25 ± 4	0.902
LGE present, n (%)	52/69 (75)	8/18 (44)	44/51 (86)	< 0.001
Ischemic-pattern, n (%)	11/69 (16)	0 (0)	11/51 (22)	
Non-ischemic patern, n (%)	41/69 (59)	8/18 (44)	33/51 (65)	

*hs cTnT* high-sensitive cardiac troponin T, *NT-proBNP*, N-terminal prohormone of brain natriuretic peptide, *LV* left ventricular, *EDV* end diastolic volume, *ESV* end systolic volume, *LVH* left ventricular hypertrophy, *ECV* extracellular volume, *LGE* late gadolinium enhancement, *T2-w SI* T2-weighted signal intensity.

*p value for comparison between hypertensive urgency and hypertensive emergency.

#### T2-weighted signal intensity ratio, T1 mapping, T2 mapping and extracellular volume fraction

The different CMR imaging biomarkers of myocardial tissue characterization are compared in Table 1 and Supplemental Table S1. Global (1.5 ± 0.2 vs. 1.4 ± 0.1, *P* = 0.044) and basal (1.5 ± 0.3 vs. 1.3 ± 0.2, *P* = 0.022) T2-w SI ratios were significantly higher in hypertensive emergency compared to hypertensive urgency. Similarly, the hypertensive crisis cohort with LVH had a higher global T2-w SI ratio than those with normal LV mass (1.5 ± 0.2 vs. 1.4 ± 0.1, *P* = 0.045). The differences observed in T2-w SI ratio remained after excluding cases of myocardial infarction from the analysis. The higher T2-w SI ratio signifies more myocardial water content in hypertensive emergency and those with LVH compared to the group with hypertensive urgency and those without LVH, respectively.

On average, the hypertensive crisis cohort (*n* = 82) had a native T1 time that was 39ms higher than the previously reported native T1 of healthy volunteers^23^ using the same scanner (1047 ± 38 ms vs. 1008 ± 31ms, *P* < 0.001). Global and basal native T1 times were numerically higher among the hypertensive emergency group compared to hypertensive urgency, but the difference was not statistically significant (*P* = 0.077 and *P* = 0.063, respectively). Similarly, Global T2 time (*P* = 0.069) and ECV (*P* = 0.051) tended to be higher in the hypertensive emergency group than in urgency. However, basal T2 time was significantly higher in hypertensive emergency (*P* = 0.004). The increased T1time, T2 time and ECV in hypertensive emergency is consistent with higher degree of myocardial edema and interstitial expansion when compared to hypertensive urgency.

**Fig. 2:**
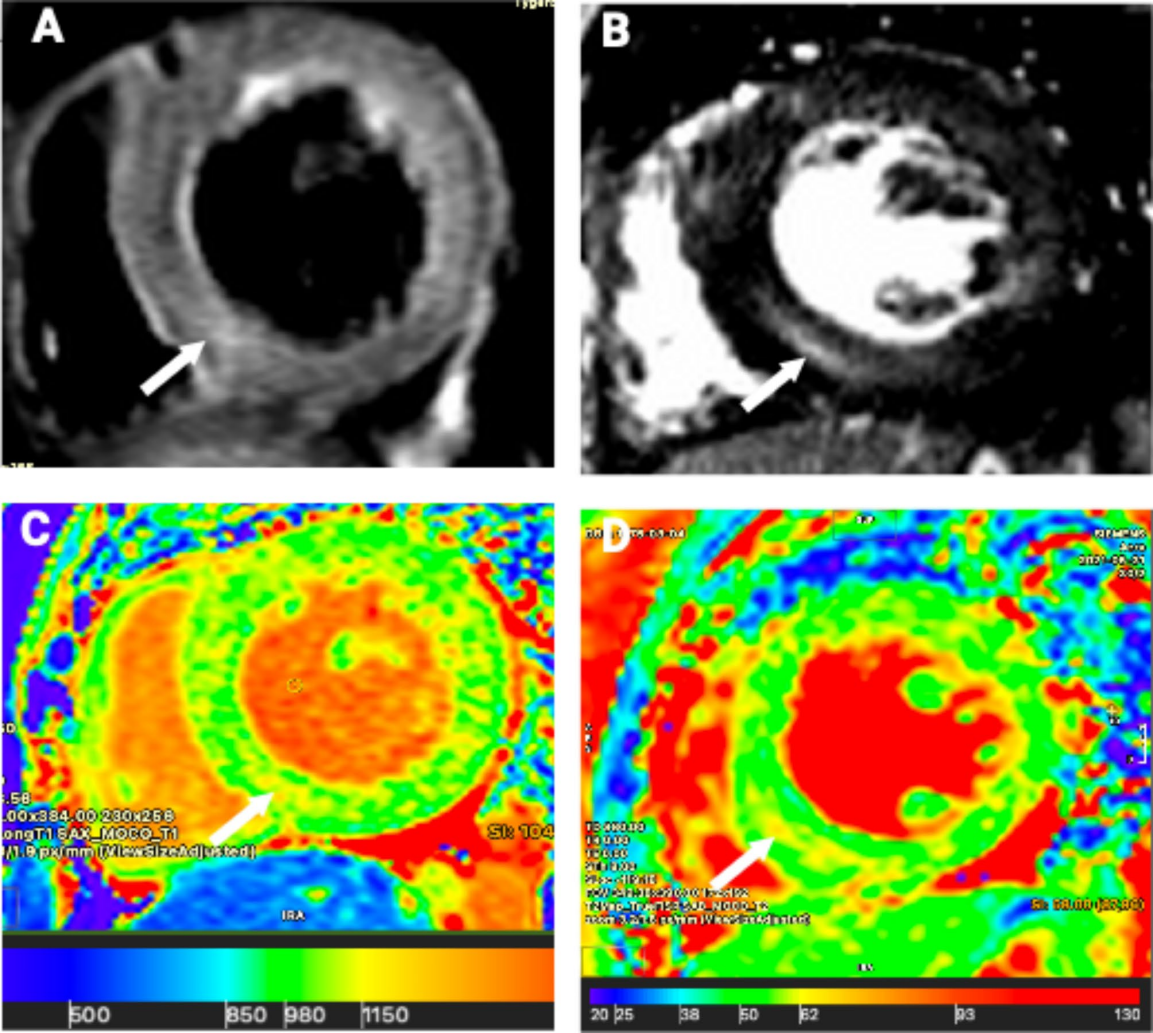
Exemplar images of myocardial oedema demosntrated using various sequences. White arrows demonstrate areas of abnormality. (**A**) Increased T2-weighted signal intensity; (**B**) non-ischaemic late gadolinium enhancement; (**C**) increased native T1; (**D**) increased T2^29^.

As illustrated in Supplemental Table S1, the hypertensive crisis cohort with LVH demonstrated a significant increase in native T1 time at all levels (*P* < 0.001), and a higher basal (*P* = 0.030) and apical (*P* = 0.034) T2 time compared to the group with normal LV mass. Similarly, ECV tended to be higher in the group with LVH (*P* = 0.05). The difference observed for the parametric mapping between hypertensive emergency and urgency persisted even after excluding cases of myocardial infarction from the analysis. Exemplar images of myocardial edema using different CMR sequences are presented in Fig. 2.

#### Subtypes of hypertensive emergency

Table 2 and Supplemental Fig. S1 shows the comparison of CMR imaging biomarkers in different types of hypertensive emergencies. T2-w SI ratio (*P* = 0.041) and T2 time (*P* = 0.026) were higher in acute pulmonary edema, signifying higher degree of myocardial edema compared to hypertensive urgency. T2 time was also noted to be higher in patients with acute pulmonary edema than those with neurological emergencies (*P* = 0.003). Although native T1 was higher in the group with acute pulmonary edema when compared to hypertensive urgency and neurological emergencies, the difference was not maintained after correction for multiple comparisons using Bonferroni method. A similar trend was observed for ECV.

**Table 2:** Comparison of cardiac magnetic resonance imaging in subtypes of hypertensive emergency.

Variable	Hypertensive urgency (*n* = 18)	Acute pulmonary oedema (*n* = 22)	Myocardial infarction (*n* = 20)	Neurological emergencies (*n* = 22)	P*
Clinical and laboratory parameters					
Age, years	48 ± 15	49 ± 14	54 ± 13	43 ± 10	0.098
Females, n (%)	13 (72)	8 (36)	7 (35)	7 (32)	0.045
Systolic blood pressure, mmHg	216 ± 26	226 ± 27	202 ± 26	224 ± 29	0.036
Diastolic blood pressure, mmHg	118 ± 16	137 ± 19	117 ± 17	136 ± 19	< 0.001
hs cTnT (ng/L)	11 (7 to 13)	64 (33 to 156)	182 (25 to 446)	17 (11 to 27)	< 0.001
NT-proBNP (ng/L)	90 (35 to 302)	1595 (530 to 5594)	336 (113 to 2513)	199 (27 to 846)	< 0.001
Cardiac magnetic resonance imaging					
Indexed LV EDV (ml/m^2^)	62 (59 to 71)	109 (90 to 140)	73 (67 to 98)	68 (61 to 88)	< 0.001
Indexed LV ESV (ml/m^2^)	21 (15 to 28)	70 (54 to 86)	33 (23 to 53)	29 (22 to 38)	< 0.001
LV ejection fraction (%)	68 ± 8	39 ± 9	55 ± 15	57 ± 13	< 0.001
Indexed LV mass (g/m^2^)	80 (66 to 95)	133 (109 to 166)	104 (82 to 134)	110 (95 to 148)	< 0.001
LVH, n (%)	8 (44)	22 (100)	15 (75)	20 (91)	< 0.001
Global T2-w SI ratio	1.4 ± 0.1	1.5 ± 0.2	1.4 ± 0.2	1.5 ± 0.2	0.037
Basal T2-w SI ratio	1.3 ± 0.2	1.6 ± 0.3	1.4 ± 0.2	1.5 ± 0.3	0.035
Midventricular T2-w SI ratio	1.4 ± 0.2	1.6 ± 0.2	1.4 ± 0.2	1.4 ± 0.2	0.061
Apical T2-w SI ratio	1.4 ± 0.2	1.5 ± 0.3	1.4 ± 0.2	1.4 ± 0.2	0.759
Global native T1 (ms)	1033 ± 40	1060 ± 30	1052 ± 41	1041 ± 38	0.156
Basal native T1 (ms)	1039 ± 39	1068 ± 26	1060 ± 40	1044 ± 33	0.040
Midventricular native T1 (ms)	1030 ± 44	1056 ± 39	1050 ± 46	1044 ± 47	0.223
Apical native T1 (ms)	1025 ± 46	1055 ± 42	1043 ± 46	1037 ± 43	0.199
Global T2 (ms)	47 ± 2	50 ± 2	49 ± 2	47 ± 2	0.002
Basal T2 (ms)	47 ± 2	49 ± 2	48 ± 2	48 ± 2	0.033
Midventricular T2 (ms)	48 ± 2	50 ± 4	50 ± 3	48 ± 2	0.013
Apical T2 (ms)	48 ± 3	50 ± 3	49 ± 3	48 ± 2	0.003
Global ECV* (%)	23 ± 4	26 ± 2	26 ± 4	23 ± 3	0.049
Basal ECV (%)	23 ± 4	26 ± 3	26 ± 4	23 ± 4	0.048
Midventricular ECV (%)	22 ± 3	26 ± 3	26 ± 5	23 ± 3	0.039
Apical ECV (%)	24 ± 7	25 ± 2	26 ± 4	23 ± 3	0.236
LGE present, n (%)	8/18 (44)	11/12 (92)	17/19 (89)	16/20 (80)	< 0.001

*hs cTnT* high-sensitive cardiac troponin T, *NT-proBNP*, N-terminal prohormone of brain natriuretic peptide, *LV* left ventricular, *EDV* end diastolic volume, *ESV* end systolic volume, *LVH* left ventricular hypertrophy, *ECV* extracellular volume, *LGE* late gadolinium enhancement, *T2-w SI* T2-weighted signal intensity.

*p value for multiple comparison.

### Correlation of imaging and cardiac blood biomarkers

**Fig. 3:**
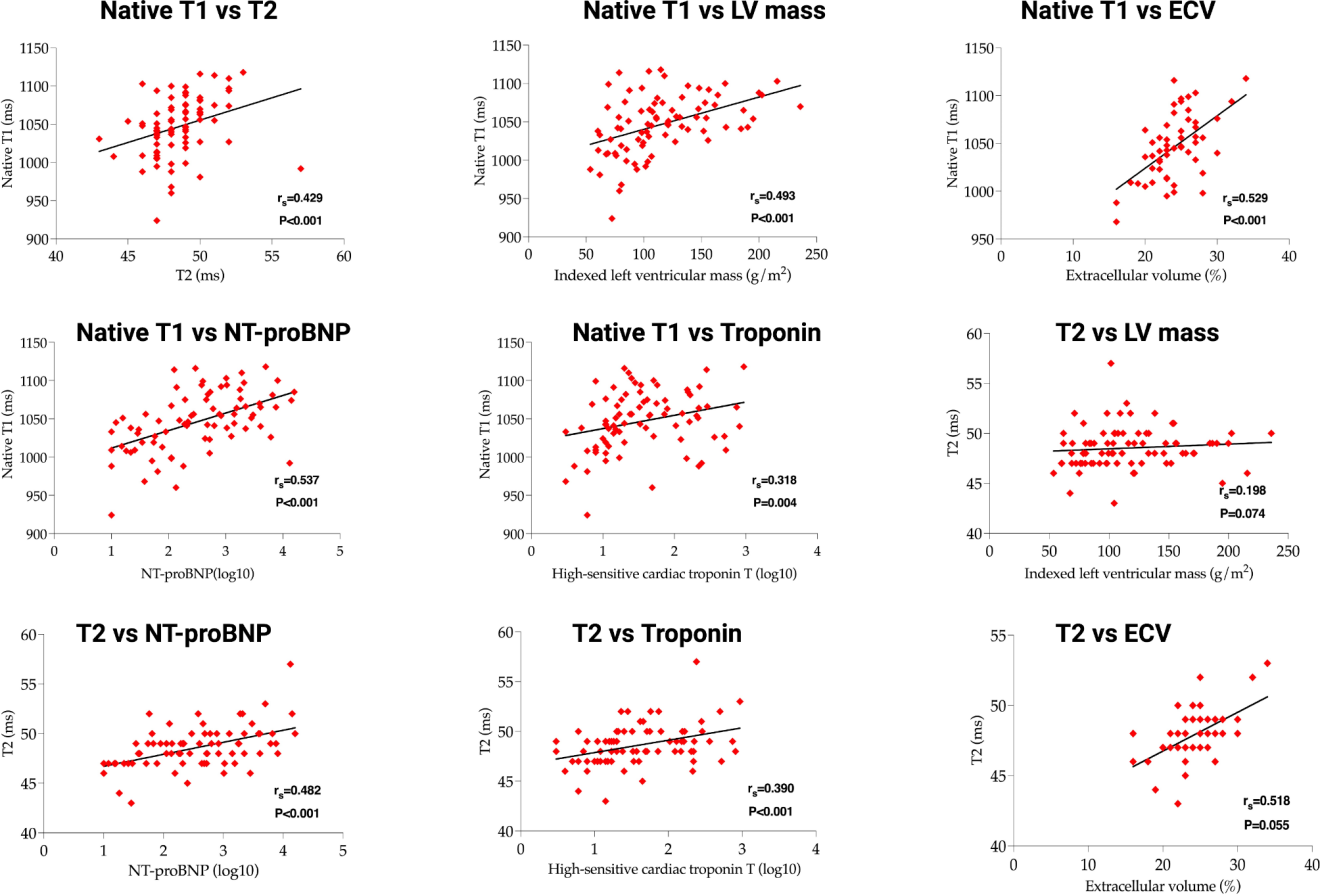
Correlation of imaging biomarkers and blood cardiac biomarkers. *ECV* extracellular volume, *LV* left ventricular, *NT-proBNP* N-terminal prohormone of brain natriuretic peptide.

The correlation between imaging and cardiac blood biomarkers is presented in Table 3 and Fig. 3. Both T1 and T2 time, as well as ECV demonstrated significant but weak correlation with hs cTnT, a marker of myocardial injury and NT-proBNP. The concordance of these CMR imaging biomarkers with cardiac troponin may be due to increased myocardial water content and interstitial expansion associated with acute myocardial injury. Although T2-w SI ratio correlated weakly with native T1, it did not show significant association with T2 time or ECV (probably due to its reduced sensitivity for myocardial edema compared to T2 time). Indexed LV mass correlated with native T1 time (*P* < 0.001) and ECV (*P* = 0.020), reflecting myocardial fibrosis and interstitial expansion associated with increasing LV mass.

**Table 3:** Correlation CMR imaging biomarkers, LV mass and serum cardiac biomarkers in patients with hypertensive crisis.

	Native T1, ms	T2, ms	T2-w SI ratio	ECV, %
Native T 1, ms	–	0.429, *P* < 0.001	0.248, *P* = 0.023	0.529, *P* < 0.001
T2, ms	0.429, *P* < 0.001	–	ns	0.518, *P* < 0.001
T2-w SI ratio	0.248, *P* = 0.023	ns	–	ns
ECV, %	0.529, *P* < 0.001	0.518, *P* < 0.001	ns	–
Indexed LV mass (g/m^2^)	0.493, *P* < 0.001	ns	0.232, *P* = 0.036	0.314, *P* = 0.020
Hs cTnT (ng/L)	0.318, *P* < 0.001	0.390, *P* < 0.001	ns	0.402, *P* = 0.003
NT-proBNP (ng/L)	0.537, *P* < 0.001	0.482, *P* < 0.001	0.223, *P* = 0.048	0.414, *P* = 0.002

*ECV* extracellular volume,*T2-w SI* T2-weighted signal intensity, *LV* left ventricle, *hs cTnT* high-sensitive cardiac troponin T, *NT-proBNP* N-terminal prohormone of brain natriuretic peptide.

### Simple linear regression and area under the receiver operator characteristics curve

Increasing hs cTnT, NT-proBNP, creatinine, and native T1 emerged as significant predictors of T2 time and myocardial edema, while increasing indexed LV mass, NT-proBNP, and T2-mapping predicted native T1 (Table 4). Using Receiver Operator Characteristic (ROC) curve, native T1 time above 1041ms predicted LVH in the hypertensive crisis cohort better than T2 (C-statistics: 0.81, *P* < 0.001 vs. 0.66, *P* = 0.043). However, native T1 or T2 could not differentiate hypertensive emergency from hypertensive urgency using ROC (C-statistics: 0.63, *P* = 0.079 vs. 0.62, *P* = 0,112).

**Table 4:** Simple linear regression analyses of factors associated with myocardial native T1 and T2.

	Native T1-time (ms)			T2-time (ms)		
	Adjusted *R*^2^	Standardized Beta	*P* value	Adjusted *R*^2^	Standardized Beta	*P* value
Age, years	0.012	− 0.110	0.327	0.062	0.248	0.025
Hs cTnT, ng/L	0.018	0.132	0.329	0.064	0.254	0.022
NT-proBNP, ng/L	0.051	0.227	0.045	0.224	0.473	< 0.001
Global T1, ms	–	–	–	0.105	0.324	0.003
Global T2, ms	0.105	0.324	0.003	–	–	–
Indexed LV mass, g/m^2^	0.200	0.448	< 0.001	0.011	0.106	0.342

*hs cTnT* high-sensitive cardiac troponin T, *LV* left ventricular, *NT-proBNP* N-terminal prohormone of brain natriuretic peptide.

### Late gadolinium enhancement

Gadolinium-based contrast (Gadovist) was administered in 69 (84%) patients (all hypertensive urgency and 80% of the hypertensive emergency group). LGE occurred in 52 (75%) patients, and this was 42% more prevalent in hypertensive emergency compared to hypertensive urgency (86% vs. 44%, *P* < 0.001, Table 1). Ischemic LGE pattern was observed in 11 (16%) patients with hypertensive emergency and myocardial infarction. Non-ischemic LGE pattern occurred in 41 (59%), and this was 21% more prevalent in hypertensive emergency than in urgency (*P* = 0.005). Compared to the group without LGE, those with non-ischemic LGE showed higher LV mass, native T1time, ECV, hs cTnT, and NT-proBNP (Supplemental Table S2). However, the T2-w SI ratio, T2 time, and LV ejection fraction did not differ between those with and those without non-ischemic LGE.

## Discussion

In this study, we utilized contrasted CMR with multi-parametric mapping to describe the myocardial tissue characteristics of patients with hypertensive crisis. The main findings of the study are as follows: (1) Native T1 time is increased in patients with a hypertensive crisis when compared to our published data on healthy volunteers (*P* < 0.001)^22^. (2) T2-w SI ratio and T2 mapping were significantly increased in the group with a hypertensive emergency. (3) Patients with hypertensive crisis and LVH had higher T2-w SI ratio and native T1 and T2 mapping than those without LVH. (4) A moderate correlation was observed between native T1 and T2, and both native T1 and T2 correlated with hs cTnT and NT-proBNP. (5) Native T1 correlated significantly with indexed LV mass and was better at predicting LVH in patients with hypertensive crisis than T2 time. In addition, LGE (predominantly non-ischemic) was observed in 75% of the hypertensive crisis cohort.

The myocardium in patients with hypertensive crisis reflects changes associated with chronic myocardial remodelling (myocardial fibrosis with or without chronic myocardial edema) and features of acute HMOD characterized by myocardial injury and increased myocardial water content. The significant increase in T2-w SI ratio and T2 mapping observed in the hypertensive emergency group and the group with LVH is consistent with myocardial edema. Increased T2-w SI ratio and T2 relaxation time reflects both acute intracellular and potentially intense interstitial edema^29^. They can occur due to an increase in absolute water content or from the uncoupling of water bound to macromolecules. However, the T2 associated with free water is 40 times longer than water bound to macromolecules^30,31^. Compared to T2w- SI, T2 mapping is less sensitive to motion artifacts and to a low signal-to-noise ratio; and provides a more reliable and quantitative measure of myocardial water^32^.

Similar to T2-w and T2 mapping, native T1 time is also sensitive to an increase in intracellular and extracellular water. However, unlike T2 mapping, increased native T1 is not specific to acute free water and can occur in other conditions associated with interstitial expansion, including myocardial fibrosis, necrosis, and infiltrative cardiac diseases^22^. Because of this lack of specificity, the increased native T1 noted in hypertensive crisis and LVH may be related to myocardial fibrosis resulting from chronic myocardial remodelling, myocardial edema associated with acute HMOD, or both. Despite the numerical variation in native T1 mapping between the hypertensive emergency and hypertensive urgency, the difference was not statistically significant. This may be related to the burden of myocardial fibrosis associated with hypertension in general or due to a type two error. In addition, this finding may reflect the fact that the association between native T1 and free water is less when compared to that of free water and T2^33^. Bohnen et al. demonstrated a better diagnostic utility of T2 for acute myocarditis than global native T1, ECV, and LGE^33^. The use of morphologic T1 and T2 STIR imaging is increasingly being supplemented, if not replaced, by T1 and T2 mapping techniques. These advanced mapping methods provide quantitative data that enhance diagnostic accuracy and reproducibility. Given these advantages, future research and clinical protocols should prioritize T1 and T2 mapping over traditional STIR imaging to improve diagnostic precision.

The significant correlations of native T1 with hs cTnT and NT-proBNP may relate the increase in native T1 to acute myocardial injury. On the other hand, the correlation of native T1 (but not T2) with indexed LV mass and diagnostic utility predicting LVH compared to T2 time (0.81, *P* < 0.001 vs. 0.66, *P* = 0.043) may imply a significant contribution of myocardial fibrosis to native T1. Kockova et al. reported a significant correlation between native T1 with indexed LV mass in patients with aortic stenosis and LVH^15^. Similarly, Fehrmann et al. showed a significant correlation between both native T1 and T2 mapping with indexed LV mass in aortic stenosis and attributed their findings to myocardial edema^14^. Like native T1, ECV is not a specific marker of a given disease and can be increased in many conditions, including interstitial myocardial edema. The significant correlations of ECV with T2, hs cTnT, and NT-proBNP in this study suggests acute myocardial injury and ischemia related to acute HMOD as the cause of myocardial interstitial expansion. Although the sensitivity of some of these imaging biomarkers may be sufficient to serve as a single marker, the complex pathophysiologic mechanisms involved in hypertensive crisis require a context-dependent interpretation for adequate myocardial tissue characterization^34^. Our study highlights the importance of ECV as an independent marker of myocardial pathology. In normal populations, ECV does not necessarily correlate with native T1 values, emphasizing its unique role in myocardial assessment. The correlation observed between ECV and clinical outcomes in our study supports the routine calculation of ECV, as recommended in recent studies^10^. Incorporating ECV measurement into standard clinical practice could significantly enhance the evaluation of myocardial health and disease.

While it is plausible to attribute edema in our cohort to myocardial infarction, the observed difference in the T2-w SI ratio and T2 time between the groups was maintained after excluding cases of myocardial infarction. Myocardial edema can occur in a hypertensive crisis for several reasons. Perivascular fibrosis, a sudden increase in LV filling pressure, reduced myocardial flow reserve, and endothelial dysfunction could worsen myocardial ischemia and result in cytogenic and vasogenic myocardial edema^35^. Secondly, increased T2 relaxation time has been reported in all forms of heart failure^36,37^. The group with acute pulmonary edema in this study had higher T2 than the groups with hypertensive urgency and neurological emergencies. We also noted a significant correlation of T2 with NT-proBNP, in keeping with the previous report^36^. In a study of postpartum patients, Joubert et al. demonstrated significant myocardial edema in patients with eclampsia and pulmonary edema using T2 mapping^38^. Despite being on the spectrum of hypertensive emergency, the pathophysiologic processes involved in eclampsia differentiate this from other forms of hypertensive emergency. It was, however, interesting to note the similar finding of myocardial edema in the setting of acute pulmonary edema, an area where limited research has been performed.

The observed alterations in CMR, such as elevated T1 and T2 values, must be interpreted with caution, as they could also be indicative of other conditions, including myocarditis. Myocarditis can present with similar non-specific myocardial changes, making differential diagnosis critical. Comprehensive clinical evaluations and additional imaging techniques including the use of LGE are essential to accurately differentiate between hypertensive crisis and myocarditis. The patients included in this study did not demonstrate LGE pattern consistent with myocarditis.

Cardiac LGE is typically observed in areas of myocardial necrosis and fibrosis and is the gold standard of non-invasive replacement fibrosis detection^39^. However, coronary vascular changes that could result in focal necrosis, reminiscent of renal fibrinoid necrosis as seen in malignant hypertension, have been demonstrated in an experimental animal model^40^. These pathophysiologic processes are aptly reflected in studies that reported concurrence of non-ischemic (replacement) fibrosis and DMF in patients with hypertension^9,41,42^. The high prevalence of non-ischemic LGE in this study may be related to the severity of hypertension and increases the risk of adverse events. It is worth noting that the increased hs cTnT and NT-proBNP (cardiac biomarkers with proven prognostic implications) noted in the group with non-ischemic LGE may point to the acuity of myocardial injury and edema, in keeping with the increased T2 mapping observed.

The differentiation between hypertensive urgency and emergency based on CMR findings has significant clinical implications. The distinct myocardial alterations observed in hypertensive emergency suggest a need for more aggressive management and closer monitoring. Future studies should continue to explore the utility of advanced CMR techniques in differentiating between these conditions and their impact on patient outcomes.

### Limitations

The findings of our study should be interpreted in the context of the following limitations. The study was conducted at Single centre (Tygerberg Hospital in South Africa) with a relatively small sample size (82 patients), which might limit the applicability of the findings to other populations or healthcare settings. A larger multicentre study could provide a more robust statistical power and enhance the external validity and applicability of the results.

The study population consists of patients referred to a tertiary hospital, which may introduce selection bias. These patients might have more severe disease or different characteristics compared to those managed in primary or secondary care settings, potentially affecting the generalizability of the results.

The study focuses on acute myocardial tissue characteristics in patients with hypertensive crisis but lacks long-term follow-up data. Longitudinal studies are necessary to understand the progression of myocardial changes and their impact on long-term cardiovascular outcomes.

A cardiac biopsy, a reference gold standard for validating the imaging findings was not done. However, numerous studies have validated the use of CMR mapping sequences for non-invasive myocardial tissue characterization.

Gadolinium-based contrast was not administered to all participants due to renal impairment, and therefore contrasted study and ECV was not obtained in some of the participants. Nonetheless, native T1 has emerged as a veritable tool for myocardial tissue characterization without the use of gadolinium-based contrast agents.

## Conclusion

Our findings demonstrate that hypertensive crisis is associated with distinct myocardial tissue alterations, including increased myocardial edema and fibrosis, as detected by CMR. Given that the differentiation between hypertensive urgency and emergency is based on clinical assessment, the role of CMR is not to distinguish between these conditions but to provide observational insights into myocardial changes. Further research is necessary to explore the prognostic value of these findings and their impact on long-term cardiovascular health.

## Electronic supplementary material

Below is the link to the electronic supplementary material.

Supplementary Material 1

## Data Availability

The data presented in this study can be obtained from the corresponding author (MAT) upon a reasonable request.
