# Supplementary material for: Characterizing myocardial edema and fibrosis in hypertensive crisis with cardiovascular magnetic resonance imaging
Source: Sci Rep. 2024 Oct 9;14:23509. doi: 10.1038/s41598-024-74099-9 (PMC11461819; doi:10.1038/s41598-024-74099-9)
Supplement: Supplementary file 1 — Supplementary Material 1 [file 41598_2024_74099_MOESM1_ESM.pdf]

Supplemental Table **S1:** Cardiac magnetic resonance compared based on with left ventricular hypertrophy.

Variable	No LVH (n=17)	LVH (n=65)	*P*
Clinical and laboratory parameters			
Age, years	48 ± 10	49 ± 14	0.886
Females, n(%)	7 (41)	28 (43)	1.000
Systolic blood pressure, mmHg	201 ± 30	222 ± 27	0.007
Diastolic blood pressure, mmHg	114 ± 9	131 ± 20	<0.001
Creatinine (µmol/L)	85 (77 to 102)	111 (88 to 174)	0.003
hscTnT (ng/L)	8 (6 to 19)	36 (16 to 152)	<0.001
NT-proBNP (ng/L)	47 (19 to 107)	534 (156 to 2077)	<0.001
Cardiac magnetic resonance imaging			
Indexed LV EDV (ml/m^2)	64 (60 to 70)	86 (66 to 109)	<0.001
Indexed LV ESV (ml/m^2)	22 (18 to 28)	38 (24 to 69)	<0.001
LV ejection fraction (%)	65 ± 7	51 ± 16	<0.001
Indexed LV mass(g/m^2)	69 (62 to 77)	118 (37 to 153)	<0.001
Global T2-w SI ratio	1.4 ± 0.1	1.5 ± 0.2	0.045
Basal T2-w SI ratio	1.4 ± 0.2	1.5 ± 0.2	0.148
Midventricular T2-w SI ratio	1.4 ± 0.2	1.5 ± 0.2	0.456
Apical T2-w SI ratio	1.4 ± 0.2	1.5 ± 0.2	0.163
Global native T1 (ms)	1013 ± 40	1056 ± 33	<0.001
Basal native T1 (ms)	1027 ± 40	1059 ± 32	<0.001
Midventricular native T1 (ms)	1006 ± 47	1056 ± 37	<0.001
Apical native T1 (ms)	1002 ± 45	1051 ± 39	<0.001
Global T2 (ms)	48 ± 2	49 ± 2	0.074
Basal T2 (ms)	47 ± 2	48 ± 2	0.030
Midventricular T2 (ms)	48 ± 3	49 ± 3	0.321
Apical T2 (ms)	48 ± 2	49 ± 3	0.034
Global ECV (%)	23 ± 3	25 ± 4	0.068
Basal ECV (%)	23 ± 4	25 ± 4	0.105
Midventricular ECV (%)	22 ± 3	25 ± 4	0.050
Apical ECV (%)	23 ± 4	25 ± 5	0.143
LGE present, n(%)	7/17 (41)	45/52 (87)	<0.001

hs cTnT, high-sensitive cardiac troponin T; NT-proBNP, N-terminal prohormone of brain natriuretic peptide; LV, left ventricular; EDV, end diastolic volume; ESV, end systolic volume; LVH, left ventricular hypertrophy; SIR, signal intensity ratio; ECV, extracellular volume; LGE, late gadolinium enhancement; T2-w SI, T2-weighted signal intensity.

Supplemental Table **S2:** Cardiac magnetic resonance findings in patients based on non-ischemic late gadolinium enhancement.

Variable	No LGE (n=17)	Non-ischemic LGE (n=41)	*P*
Age	47.4(14.2)	48(14.1)	0.817
Females	11 (65)	17 (42)	0.093
Systolic blood pressure	207 ± 23	222 ± 28	0.092
Diastolic blood pressure	122 ± 18	131 ± 20	0.067
Creatinine (µmol/L)	77 (60 to 92)	108 (88 to 119)	<0.001
hs cTnT (ng/L)	8(5 to 17)	21 (13 to 39)	0.001
NT-proBNP (ng/L)	5 (15 to 151)	396 (113 to 1037)	0.001
Indexed LV EDV, mL/m^2	62 (58 to 70)	74 (63 to 100)	0.009
Indexed LV ESV, mL/m^2	23 (17 to 31)	31 (21 to 53)	0.069
LV ejection fraction, %	61 (55 to 71)	58 (45 to 67)	0.191
Indexed LV mass, g/m^2	75 (62 to 93)	111 (96 to 142)	<0.001
Maximum LV wall thickness (mm)	14 ± 1.8	16 ± 3.6	0.001
Global T2-w SI ratio	1.4 ± 0.2	1.5 ± 0.2	0.140
Basal T2-w SI ratio	1.4 ± 0.2	1.5 ± 0.3	0.131
Midventricular T2-w SI ratio	1.4 ± 0.2	1.5 ± 0.2	0.111
Apical T2-w SI ratio	1.4 ± 0.2	1.5 ± 0.3	0.230
Global native T1 mapping (ms)	1015 ± 41	1055 ± 30	<0.001
Basal native T1 mapping (ms)	1019 ± 35	1059 ± 29	<0.001
Midventricular native T1 mapping (ms)	1012 ± 48	1056 ± 35	<0.001
Apical native T1 mapping (ms)	1011 ± 49	1048 ± 35	0.002
Mid septal native T1 mapping (ms)	1024 ± 40	1049 ± 48	0.067
Global T2 mapping (ms)	48 ± 2	48 ± 2	0.259
Basal T2 mapping (ms)	47 ± 2	48 ± 2	0.101
Midventricular T2 mapping (ms)	48 ± 2	48 ± 2	0.706
Apical T2 mapping (ms)	48 ± 2	48 ± 2	0.239
Mid septal T2 mapping (ms)	48 ± 5	49 ± 5	0.330
Global ECV (%)	22 ± 3	24 ± 3	0.004
Basal ECV (%)	21 ± 4	25 ± 3	0.003
Midventricular ECV (%)	22 ± 4	24 ± 2	0.008
Apical ECV (%)	22 ± 4	25 ± 5	0.035

hs cTnT, high-sensitive cardiac troponin T; NT-proBNP, N-terminal prohormone of brain natriuretic peptide; LV, left ventricular; EDV, end diastolic volume; ESV, end systolic volume; LVH, left ventricular hypertrophy; ECV, extracellular volume; LGE, late gadolinium enhancement; T2-w SI, T2-weighted signal intensity.

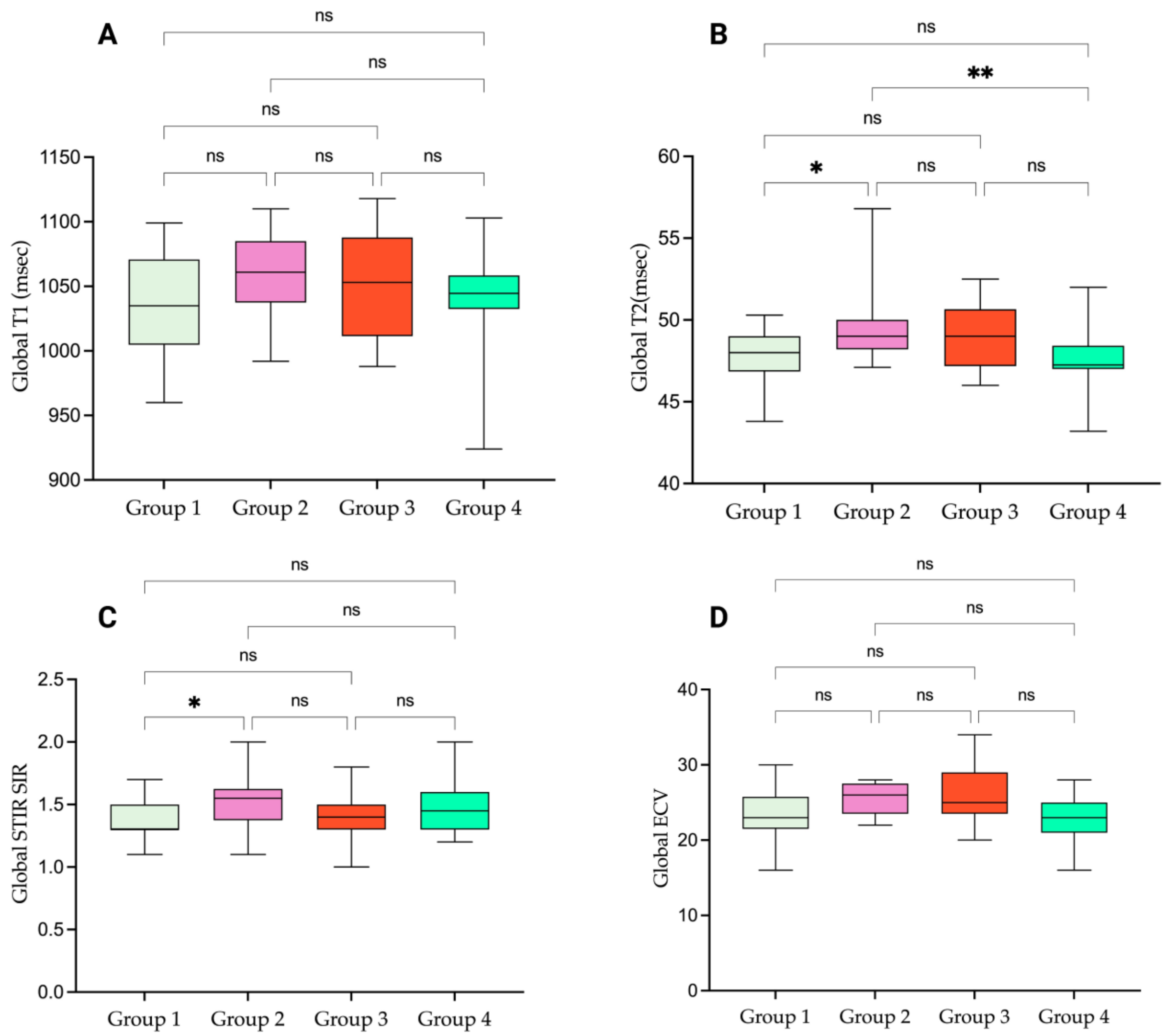

Supplemental Figure **S1**: Comparison of imaging biomarkers in subtypes of hypertensive emergency. A) Global T1; B) Global T2 (**P*=0.026, ***P*=0.003); C) Global single tau inversion recovery (STIR) signal intensity ratio (SIR) (**P*=0.045); D) Global extracellular (ECV) fraction. Group 1, hypertensive urgency; Group 2, acute pulmonary oedema; Group 3, myocardial infarction; Group 4, neurological emergencies.
